# Dual encoder–decoder-based deep polyp segmentation network for colonoscopy images

**DOI:** 10.1038/s41598-023-28530-2

**Published:** 2023-01-21

**Authors:** John Lewis, Young-Jin Cha, Jongho Kim

**Affiliations:** 1 Department of Civil Engineering, University of Manitoba, Winnipeg, R3M 0N2 Canada; 2 Department of Radiology, Max Rady College of Medicine, University of Manitoba, Winnipeg, R3A 1R9 Canada

**Keywords:** Colonoscopy, Software

## Abstract

Detection of colorectal polyps through colonoscopy is an essential practice in prevention of colorectal cancers. However, the method itself is labor intensive and is subject to human error. With the advent of deep learning-based methodologies, and specifically convolutional neural networks, an opportunity to improve upon the prognosis of potential patients suffering with colorectal cancer has appeared with automated detection and segmentation of polyps. Polyp segmentation is subject to a number of problems such as model overfitting and generalization, poor definition of boundary pixels, as well as the model’s ability to capture the practical range in textures, sizes, and colors. In an effort to address these challenges, we propose a dual encoder–decoder solution named Polyp Segmentation Network (PSNet). Both the dual encoder and decoder were developed by the comprehensive combination of a variety of deep learning modules, including the PS encoder, transformer encoder, PS decoder, enhanced dilated transformer decoder, partial decoder, and merge module. PSNet outperforms state-of-the-art results through an extensive comparative study against 5 existing polyp datasets with respect to both mDice and mIoU at 0.863 and 0.797, respectively. With our new modified polyp dataset we obtain an mDice and mIoU of 0.941 and 0.897 respectively.

## Introduction

Colorectal cancer (CRC) is a form of cancer that impacts the large intestine, constituting one of the most severe and most common forms of cancer. 5-year survival rates depend on a variety of different factors and can vary significantly depending on the stage and whether the cancer is primarily located in the colon or the rectum. Average 5-year survival rates for CRC are estimated to be at 48.6% to 59.4%^1^. These cancers often begin as small, benign tumors referred to as polyps that over time will become cancerous. Commonly referred to as adenomas, the early detection and removal of these tissues is essential.

Polyps are imaged through an invasive procedure known as colonoscopy. This procedure involves moving a camera through the digestive tract to gather image data on the state of the digestive system and identify potential polyps. This imaging process has been recognized as the landmark procedure in reducing the incidence rate of CRC. A.G. Zauber et al.^2^ demonstrate that a 53% reduction in mortality can be achieved using this imaging procedure to detect polyps. Amidst promising results in detection of polyps within the digestive tract, the procedure itself is subject to human error. The miss rates of polyps imaged through back-to-back colonoscopies can reach up to ~ 15–30% depending on the size of the polyp as reported by^3^.

With initial work in convolutional neural networks (CNNs) pioneered by^4^, today, the vast majority of segmentation models using CNN-based architectures feature an encoder–decoder based structure, as seen in^5,6^, and^7^. In these networks an image is progressively processed with various convolutional and other mathematical operations to lower-level feature maps, and thus local, short-range semantic information is extracted. It is well understood that a major shortcoming of CNNs is their inability to extract global dependencies due to the local nature of the convolutional operation, reflective of the inherent trade-off between model speed and complexity.

Common datasets appearing in the evaluation of polyp detection and segmentation networks are the Kvasir-SEG dataset provided by^8^, the CVC-ClinicDB dataset provided by^9^, the CVC-ColonDB dataset provided by^10^, the ETIS dataset provided by^11^, and the EndoScene dataset provided by^12^. A variety of different CNN-based approaches have been taken with respect to these datasets, such as with^13^ who developed a segmentation model using a 3-dimensional, fully convolutional (3D-FCN) network. This model achieved state-of-the-art (SOTA) performance in terms of its F1-Score and F2-Score. In^14^ an FCN network was developed with a unique structure in which initial predictions were first made using binary classification and then fed through a CNN similar to UNet as designed by^7^. This network achieved SOTA performance in terms of the Kvasir-SEG and the CVC-ClinicDB dataset with respect to sensitivity and specificity metrics.

Other issues endemic to CNNs beyond capturing global dependencies, are overfitting and the ability to capture boundary pixel information with adequate accuracy. More recently, there have been explicit attempts to address these issues, such as with PraNet, developed by^15^. This model proposed real-time segmentation capabilities by exploiting deep supervision mechanisms and boundary detection with a reverse attention module, as well as employing a parallel-partial decoder. These modules have been also found in different novel architectures such as AMNet, by^16^, which further improves upon edge-detection capabilities harnessed by PraNet.

Other recent developments in polyp segmentation, such as with FANet by^17^, have shown novel approaches to attention and refining predictions based off coarser representations. More specifically, the authors utilized information across training epochs to make better predictions across learnable parameters in subsequent epochs, providing a unique form of attention. The model produced excellent results with the CVC-ClinicDB dataset, however its results with respect to the Kvasir-SEG datasets were not SOTA. Another novel approach to attention in which multiple-resolution scales were uniformly fused throughout the model was shown in GMSRF-Net^18^. Other multi-scale approaches have been found in MSNet^19^ which showed positive results with respect to polyp segmentation, however the results have since been improved upon. In^20^, a novel approach was presented in which a color exchange structure was presented to help the model focus on the structure of the polyp and filter out noise associated with color. Good results were presented however the presented metrics have since been surpassed.

Ensemble methods, like dual-encoder and/or dual-decoder architectures have appeared over recent years, such as with^21^ and ^22^. In^21^, the dual encoder–decoder showed good results with respect to polyp segmentation, however the dual model structure was applied in sequence, as opposed to synchronously, and the result of one encoder–decoder was used as input for the following encoder–decoder segment. Moreover, there were little-to-no novel additions to the network, as all components were existing, pretrained architectures. In^22^, a dual decoder network was presented in DDANet, where a single ResNet-style encoder was used with a dual-decoder architecture in which both a grayscale image and a segmentation mask were generated by each decoder. While the approach was creative, significant improvements on the metrics generated by the network have been generated since. In addition to recent advances in ensemble methods, there have also been advances in transformers within the field of computer vision, as seen in^23–26^, and^27^.

Other ensemble methods, specifically dual-model approaches have also been observed in an effort to increase accuracy of output segmentation maps detailing the pixel-level location of polyps. Examples of this have been observed with polyp segmentation networks such as the dual mask R-CNN model as seen in^28^ and the dual DeiT transformer and ResNet CNN structure proposed by^29^. Ali et al.^30^ took a general approach and evaluated a variety of different existing CNN-based architectures capable of segmentation and classification of endoscopic objects. Other means of improving upon the shortcomings of CNN-based methods have been explored. Of particular importance to the field of study with this paper is the utilization of deep supervision and its impact on the field of polyp segmentation, and more generally, medical image segmentation. As seen in^15,29,31^, and^32^, to varying degrees, each model generates several output segmentation maps from various coarser representations which contribute directly to the final output segmentation map, allowing the model to converge more quickly to an optimum.

In this paper, we propose a novel DL-based architecture, PSNet based on the corresponding authors’ extensive experiences and knowledge with SOTA performances in deep learning networks, as shown in^33^ and^34^. As well as in detection and segmentation problems as shown in^35–37^, and^38^. To improve upon the boundary pixel limitations of previous polyp segmentation networks and to improve upon overall polyp segmentation capabilities, we have designed PSNet, a unique dual-model architecture, an encoder–decoder-based end-to-end network. PSNet is composed of a dual encoder and dual decoder. The dual encoder consists of a novel encoder, referred to as PS encoder, as well as a transformer encoder from^39^. The dual decoder consists of the PS decoder, a partial decoder, an enhanced dilated transformer decoder, as well as a sequence of merge modules that each produce candidate segmentation maps. The PS encoder, PS decoder, enhanced dilated transformer decoder and merge module are all newly developed for this original research study. We first discuss the methodology of PSNet, followed by presentation of results on leading datasets in polyp segmentation, and then follow it with a discussion.

## Methodology

Our research paper proposes a new dual encoder–decoder structure, referred to as PSNet, to segment polyps on a pixel-based level. We have developed several novel modules for the purpose of polyp segmentation. These are the PS encoder, PS decoder, the merge module, along with their relevant components, the local feature extraction (LFE) module and the dual complex convolutional module (CCM). And to achieve SOTA performance of the polyp segmentation network, we carefully integrate these previously mentioned components with existing modules such as a ViT-based transformer encoder and partial decoder within the dual encoder–decoder-based structure.

**Figure 1:**
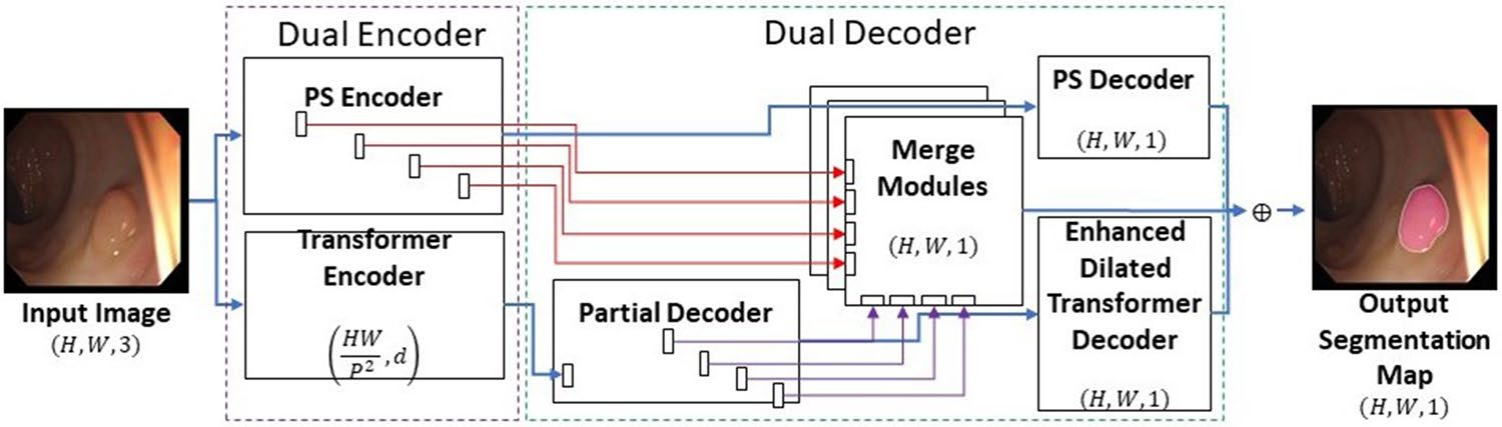
Overall schematic view of our proposed PSNet.

The image input size for the network is designed at $(H =$ 512) × $(W =$ 512) and takes in RGB images/videos produced from endoscopic procedures as shown in Fig. 1. PSNet is thus capable of identifying on a pixel-based level the location of polyps and distinguishing them from healthy tissue. The dual encoder structure consists of a novel CNN-based encoder, referred to as the PS encoder, which runs synchronously with a transformer-based encoder as shown in Fig. 1. Following the feature extraction and dimension reduction in the encoder, the input feature maps are processed through a dual decoder structure, which consists of our own novel PS decoder, merge modules, and enhanced transformer decoder. A partial decoder is used to transform the output from the transformer encoder to 4-dimensional (D) input. Attention is incorporated at almost every level and module in the network, through skip connections between the PS encoder and PS decoder, through the attention mechanism in the transformer encoder, as well as through the candidate segmentation maps produced by the merge modules.

The motivation behind generating a dual model structure using CNN-based approaches and transformers lies in the fact that transformers distinguish themselves at being apt at gathering global contextual information. Conversely, CNNs are inherently limited to local level feature information due to the inherent limitations of the convolutional operation. Thus, the primary motivation behind combining the two distinct architectures was the ability to fuse both of these design features into one. The two structures work reciprocally by having one component of the network focus primarily on global relationships between pixels and the other focuses on extracting local or smaller-scale feature information. Capturing global dependencies is fundamentally a more expensive operation. Consequently, the transformer is the heavier component in terms of model complexity.

The PS decoder, the enhanced dilated transformer decoder, and the merge modules produce candidate output segmentation maps and are averaged on a pixelwise level to produce the final outputs. Using deep supervision, merge modules exist between the transformer encoder and the PS encoder which integrates the intermediate representations. The merge modules harness the PS encoder’s and the transformer encoder’s respective focuses on local and global information, respectively, and when working synchronously with each other, model complex relationships and help improve the model accuracy. These modules and their respective input paths are indicated with the red and purple lines in Fig. 1. The details of the developed blocks are described in the following subsections.

### Dual encoder

Our newly developed dual encoder structure is a combinatory structure composed of both a CNN-based encoder and a transformer encoder, the VisionTransformer (ViT)^39^. Each encoder works on specific regions and scales. The two encoders work synchronously and produce outputs that are integrated at multiple scales in the decoder structure.

#### PS encoder

**Figure 2:**
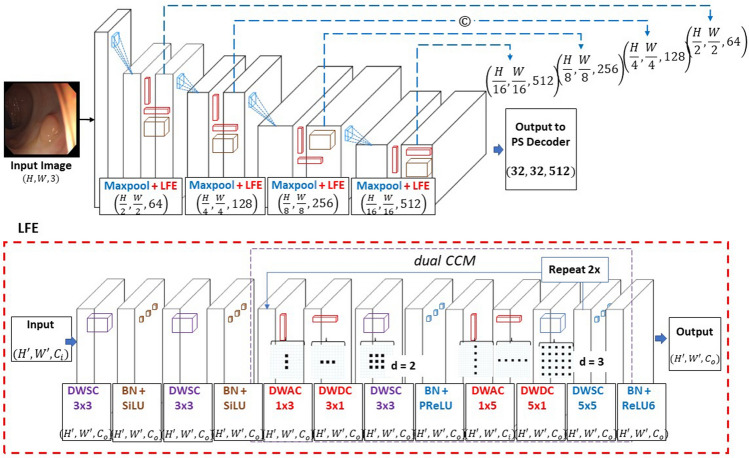
Details of the proposed PS encoder with LFE module.

The PS encoder is a newly developed CNN encoder that is designed to extract local multilevel features efficiently. The overall schematic of PS encoder is presented in Fig. 2. The PS encoder takes in the same input as the transformer encoder and puts it through a sequence of four identical maxpooling operations, each followed by our newly developed LFE module. The purpose of the maxpooling operation being to reduce the spatial dimensions and to highlight the features of primary importance. Each LFE module consists of a dual depthwise separable standard convolution (DWSC), followed by a CCM submodule. The LFE module as shown in Fig. 2 is the foundational block of the PS encoder and within the module itself uses a combination of both standard and dilated convolutions to gather rich contextual information in multilevel local features. The encoder takes in an input, $\mathrm{x}\in {\mathrm{R}}^{\mathrm{H}\times \mathrm{W}\times 3}$, and produces an output, $\mathrm{x}\in {\mathrm{R}}^{\frac{\mathrm{H}}{16}\times \frac{\mathrm{W}}{16}\times 512}$, after which it proceeds to the PS decoder.

The primary purpose of the LFE module is to maximize the local level feature extraction, while increasing the scope of the local level feature extraction process. This is done by increasing the receptive fields in convolutional operations throughout the module. At the start of the LFE module, the input feature map is put through the two DWSCs with a square kernel size of 3, and a stride and padding of 1. The primary design motivation behind using a relatively small kernel size along with a unit dimension stride and padding was the notion that larger reductions in the spatial dimension of the output feature map can potentially result in information loss. Kernel sizes are kept small to focus on local features.

The CCM is composed of a depth-wise asymmetric convolution (DWAC), followed by a depthwise dilated convolution (DWDC). The CCM is newly designed, inspired by^38^. The key role of the dual CCM submodule is to learn complex correlations between local features that are integral to the input. The progression follows a general path of $1\times \mathrm{k}$, then $\mathrm{k}\times 1$, then $\mathrm{k}\times \mathrm{k}$, with $\mathrm{k}$ initially being 3, and then 5 in the second iteration of convolutions. Moreover, the dilation rate is modified throughout the module, with $\mathrm{d}=2$ for the $\mathrm{k}=3$ convolutions and then $\mathrm{d}=3$ in the second sequence for the $\mathrm{k}=5$ convolutions. Batch normalization follows all convolutional layers and two activation functions are applied at the end of each set of $\mathrm{k}=3, 5$ convolutions. Since each of the CCM submodule’s convolutional kernels have a wider receptive field, broader and more complex feature information can be extracted. Thus, as a corollary benefit to the local feature extraction, the dual CCM module also obtains the relative global spatial features that are integral to the boundary regions of the polyp. Given that separable convolutions are used throughout the CCM submodule, each module improves the computational complexity and performance of the model.

After each convolution in the LFE module, batch normalization (BN) and one of 3 atypical activation functions are implemented. These 3 functions being the sigmoid linear unit (SiLU), the parametric rectified linear unit (PReLU), and the rectified linear unit 6 (ReLU6) activation function^40^. Each DWSC before the dual CCM is followed by the SiLU activation. The SiLU activation is similar in shape to the ReLU, the latter of which is frequently implemented into CNN architectures due to its relative simplicity in backpropagation calculations, as well as its lower computational cost. However, the SiLU function has a smoother curve shape relative to the ReLU function, while maintaining stability in training. In designing the architecture, we noticed an improved performance when substituting the nonlinear activation functions in the PS encoder from ReLU to SiLU.PReLU is chosen because it can often provide better accuracy and perform better in terms of saturation. Moreover, the PReLU also employs a learnable parameter which can further improve model performance. We select ReLU6 for the second activation at the end of the module. The ReLU6 function is identical in shape to the ReLU function, however the activation has a maximum output value of 6. This contributes to increased robustness when utilized with lower precision calculations.

#### Transformer encoder

**Figure 3:**
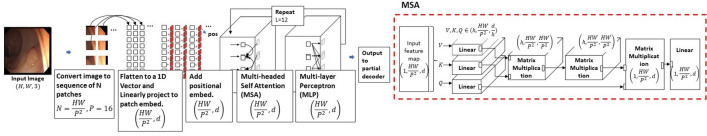
Details of the transformer encoder with MSA module.

The transformer encoder is a transformer-based (ViT) architecture^41^. A schematic of the transformer encoder is presented in Fig. 3.

It takes an input image, which is then patched, flattened, and converted to a sequence of patch embeddings. These embeddings are then fed through its main self-attention mechanism, which consists of a sequence of multi-scale self-attention (MSA) modules and a multi-layer perceptron (MLP). This attention mechanism is repeated *L* times before returning the intermediate feature map to the decoder. The primary purpose of the transformer encoder is to learn long-range dependencies by explicitly learning the correlation between a pixel (or higher-dimensional representation of such a pixel), with respect to all other locations in the extracted features, which is achieved through the self-attention mechanism.

### Dual decoder

The architecture of the dual decoder consists of two synchronous decoders, PS decoder and the transformer decoder, and a synchronous set of merge modules, as seen in Fig. 1. The transformer decoder consists of a partial decoder which reshapes the input from the transformer encoder, followed by our novel dilated convolutional decoder, referred to as the enhanced dilated transformer decoder. The primary purpose of the PS decoder is to focus on local level feature information and decoding the output from the PS encoder to a candidate output segmentation map. The purpose of the partial decoder is to convert the output of the transformer encoder from 2 to 3D. The purpose of the enhanced transformer decoder is to convert this output to a candidate segmentation map while retraining the global level feature information extracted from the transformer encoder. The purpose of the merge modules is to provide a set of additional candidate segmentation maps from the integration of coarser representations present in the PSNet dual encoder and reinterpret this into actual semantic information to further compensate for feature loss endemic to CNNs and transformers.

#### PS decoder with wSq module

**Figure 4:**
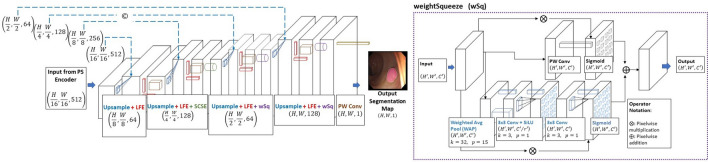
Details of the proposed PS decoder and wSq module.

Similar to our PS encoder, our novel PS decoder as shown in Fig. 4 consists of a sequence of upsamplings, LFE modules, concurrent channel and spatial squeeze and excitation modules (SCSE) as shown in^42^ and our own novel modification to these SCSE modules, which we refer to as weighted concurrent channel and spatial squeeze and excitation modules (wSq) modules. The primary purpose of the PS decoder is to focus on local level feature information and decoding the output from the PS encoder to a candidate output segmentation map.

The input feature map, $\mathrm{x}\in {\mathrm{R}}^{\frac{\mathrm{H}}{16}\times \frac{\mathrm{W}}{16}\times 512}$, received from the encoder is fed into the first module of the PS decoder, which is an upsampling operation and LFE module (see Fig. 2). This process is repeated 3 more times with either SCSE modules or wSq modules applied following the LFE module execution, as shown in Fig. 4. The final operation is a PW convolution to generate an output segmentation map, $\mathrm{x}\in {\mathrm{R}}^{\mathrm{H}\times \mathrm{W}\times 1}$. Two wSq modules and one SCSE module are used in the PS decoder. The wSq module operates by extracting global spatial information using what we refer to as a weighted average pooling operation (WAP) in its spatial squeeze component, sSE, as seen in Fig. 4.

The sequence of operations is as follows: Firstly, the input signal goes through the weighting function, WAP, to extract global spatial information, then the feature map in its condensed form goes through two PW convolutions, followed by a sigmoid activation which acts as a smooth gating function. The output from this is multiplied by the input and produces the output for the spatial squeeze components. Concurrently, the input is also put through a channel-wise squeeze component, cSE, in which the original input signal is put through a PW convolution and a subsequent sigmoid activation. The entire operation is summarized in Eq. (1).

1 $$\mathrm{y}=\mathrm{x}*\mathrm{cSE}\left(\mathrm{x}\right)+\mathrm{x}*\mathrm{sSE}(\mathrm{x})$$

where, x is the input signal to the module, cSE is the spatial squeezing component, and sSE is the channel-wise squeezing component. Through parametric studies, the chosen padding and kernel dimensions utilized in the WAP contributed the most to long-term retention of global feature information. Intuitively this makes sense with respect to the fact that the larger padding size and larger kernel size allows for larger regions of focus output from the pooling layer.

#### Partial decoder, enhanced dilated transformer decoder and merge module

**Figure 5:**
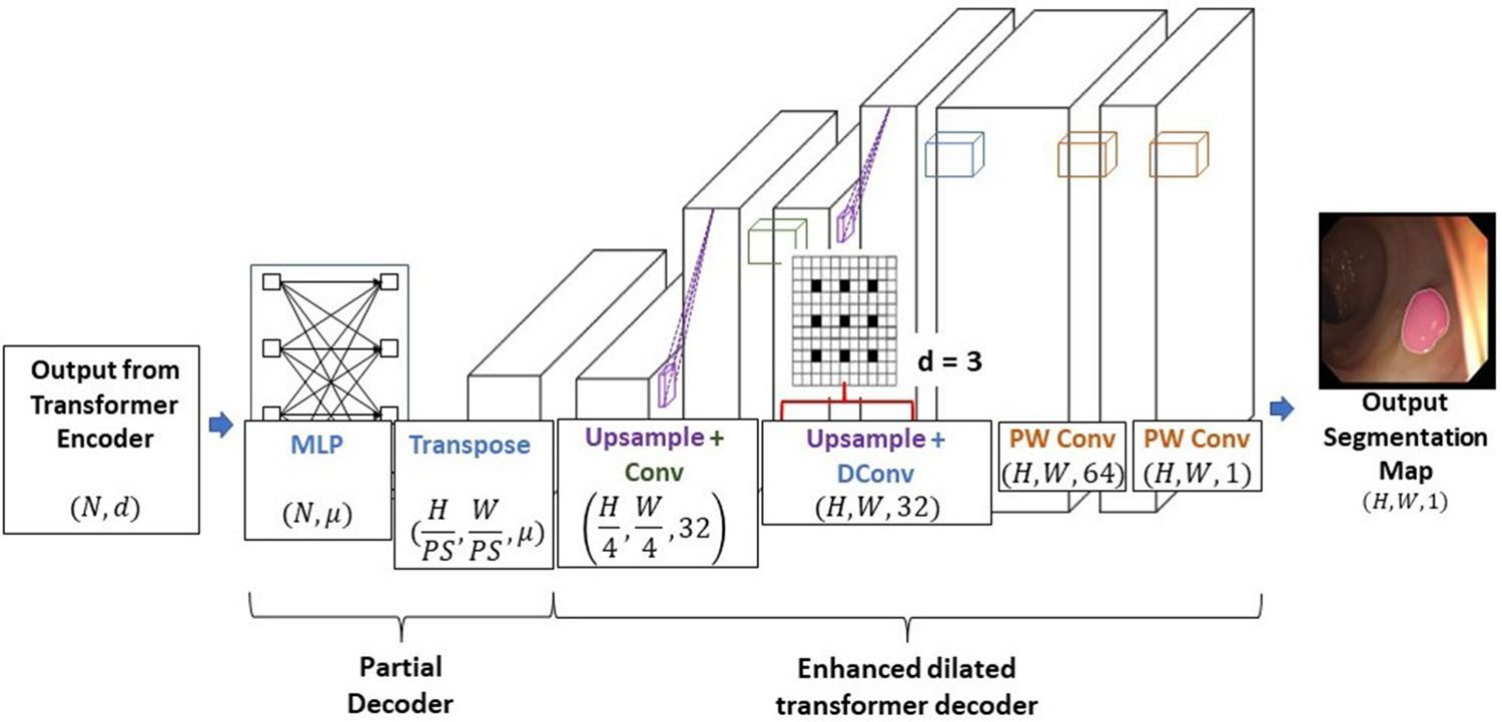
Details of the partial decoder and proposed enhanced dilated transformer decoder.

The partial decoder is newly developed to receive the feature map data from the transformer encoder, shown in Fig. 1, and its ultimate purpose is to convert the output it receives from 2 to 3D. Based on the decoder provided by^27^, the partial decoder receives a sequence of patch encodings containing rich semantic information in 2D form, $\mathrm{x}\in {\mathrm{R}}^{\mathrm{N}\times \mathrm{d}}$, where N is the number of patches and $\mathrm{d}$ is the embedding dimension. This input is then put through a pointwise linear layer which generates a list of probabilities or logits, with the output being $\mathrm{x}\in {\mathrm{R}}^{\mathrm{N}\times\upmu }$, where $\upmu$ is the number of logits as shown in Fig. 5. The enhanced dilated transformer decoder is a simple dilated convolutional-based decoder and is also presented in Fig. 5.

We treat $\upmu$ in our design as a hyperparameter, where each logit represents a different type of tissue and select $\upmu =64$. In doing this, we allow for our model to be able to distinguish between different conditions of polyp pixels (boundary pixels, pixels with specific lighting conditions, etc.) that are present in the image, and allow for a higher model accuracy and better performance. The second and final operation in the partial decoder is a transpose function which rearranges the input tensor from 2 to 3D. The function used is the rearrange function from the einops library. With this function, the feature map is reshaped to a coarse representation of the output segmentation map at 1/16th the original input size, $\mathrm{x}\in {\mathrm{R}}^{\frac{\mathrm{H}}{16}\times \frac{\mathrm{W}}{16}\times\upmu }$. This feature map then proceeds to our proposed enhanced dilated transformer decoder.

The purpose of the enhanced transformer decoder is to convert this output to a candidate segmentation map while retraining the global level feature information extracted from the transformer encoder. It consists of upsampling and a double standard convolution, followed by upsampling and a double dilated convolution, followed by two pointwise convolutions. The final output is a candidate segmentation map with dimensions $\mathrm{x}\in {\mathrm{R}}^{\mathrm{H}\times \mathrm{W}\times 1}$. Dilated convolutions are chosen in the decoder given their ability to extract more global feature information with their larger receptive field, and thus improve on even further, the capacity for the transformer branch to extract global feature information. The dilation rate, r, was chosen to be 3 as indicated in Fig. 5. While dilated convolutions are known to potentially contribute to information loss, we found improvement in our own analyses that dilated convolutions further improved on the model performance when compared to the entirely standard convolution implementation of the decoder.

The merge module amalgamates the outputs from both the PS encoder and the feature maps received from the partial decoder, which is correspondingly received from the transformer encoder. The primary purpose of the merge module is to integrate the features between the PS encoder, which focus on local phenomena, and the transformer encoder which focuses on global dependencies. Integration of features between the PS encoder and the transformer occur at multiple different feature scales through the merge module.

The number of channels throughout the merge module is kept constant at 64 to reduce computational overhead. In terms of operation sequence, the outputs from the transformer and the PS Encoder are first concatenated along the channel dimension. The concatenated input feature map is then put through two maxpooling operations, each followed by a double DWSC with BN and SiLU. The feature map is then put through two upsamplings, each followed by the same DWSC sequence, and then upsampled to generate a candidate segmentation map.

### Datasets and training details

In this section, the datasets used to evaluate PSNet’s performance are presented, along with how they were configured in terms of training, testing, and validation divisions. The specific training details of the model are also presented, such as the optimizer and learning rate scheduler used to update the learnable parameters in the model.

#### Datasets

An Intersection-over-Union (IoU) loss function is used to train the model^43^, in which six candidate output segmentation maps are averaged prior to entry into the loss layer. This loss function is summarized in Eq. (2). The final output segmentation map arrangement is summarized in Eq. (3).

2 $${\mathrm{L}}_{\mathrm{n}}= \frac{\left|\upsigma \left(\overline{{\mathrm{x} }_{\mathrm{n}}}\right)\cap {\mathrm{y}}_{\mathrm{n}}\right|}{\left|\upsigma \left(\overline{{\mathrm{x} }_{\mathrm{n}}}\right)\cup {\mathrm{y}}_{\mathrm{n}}\right|}$$

3 $$\overline{{\mathrm{x} }_{\mathrm{n}}}=\frac{{{\mathrm{x}}_{\mathrm{n}}}^{\mathrm{T}}+{{\mathrm{x}}_{\mathrm{n}}}^{\mathrm{C}}+{{\mathrm{x}}_{\mathrm{n}}}^{1/16}+{{\mathrm{x}}_{\mathrm{n}}}^{1/8}+{{\mathrm{x}}_{\mathrm{n}}}^{1/4}+{{\mathrm{x}}_{\mathrm{n}}}^{1/2}}{6}$$

where, ${\mathrm{L}}_{\mathrm{n}}$ is the IoU loss, $\upsigma$ is the sigmoid activation function and $\overline{{\mathrm{x} }_{\mathrm{n}}}$ is the averaged output segmentation map produced from the mean of the 6 candidate output segmentation map streams (i.e. ${{\mathrm{x}}_{\mathrm{n}}}^{\mathrm{T}}+{{\mathrm{x}}_{\mathrm{n}}}^{\mathrm{C}}+{{\mathrm{x}}_{\mathrm{n}}}^{1/16}+{{\mathrm{x}}_{\mathrm{n}}}^{1/8}+{{\mathrm{x}}_{\mathrm{n}}}^{1/4}+{{\mathrm{x}}_{\mathrm{n}}}^{1/2})$, the output of $\upsigma \left(\overline{{\mathrm{x} }_{\mathrm{n}}}\right)$ is the final output segmentation map, and ${\mathrm{y}}_{\mathrm{n}}$ is the ground truth corresponding to the input image of the network.

A total of 5 publicly available datasets are used to either train or evaluate the model. The Kvasir-SEG^8^, CVC-ClinicDB^9^, CVC-ColonDB^10^, ETIS^11^, and EndoScene^12^ datasets are used to evaluate the model. The Kvasir-SEG dataset consists of 1000 images ranging in size between 487 × 332 and 1920 × 1072. These images were collected from Vestre Viken Health Trust in Norway. The images were manually labelled by a medical doctor and the resulting annotations were validated by an experienced gastroenterologist. The CVC-ClinicDB dataset consists of 612 images of size 384 × 288. These images were collected from 29 polyp video sequences from a total of 23 patients, and were labelled by expert gastroenterologists. The CVC-ColonDB dataset consists of 380 images of size 574 × 500. These images were collected from a total of 12,000 images from 15 short colonoscopy videos, of which only 380 were annotated. The images were carefully selected for annotation to distinguish a wide range of colors, textures, and features. The ETIS dataset consists of 196 images of size 1225 × 966 from wireless capsule (WCE) endoscopic images. These images were collected by the authors from the Universitat Autonoma de Barcelona from an original total of 300 endoscopy images and were labelled by expert gastroenterologists. The EndoScene dataset consists of 60 images of size 574 × 500. The images in the EndoScene dataset are a combination of the CVC-ClinicDB dataset and the CVC-300 set, which exclude the repeated images and thus represent a unique dataset. The dataset was produced from a total of 44 video sequences from 36 patients.

In order to validate the performance of the proposed PSNet, we follow the same training and testing procedures that other competitive models use, such as in^15,16^, and^29^, which use the Kvasir-SEG, CVC-ClinicDB, CVC-ColonDB, ETIS, and EndoScene datasets. The general approach has been to train the model on a combined training dataset consisting of the Kvasir-SEG dataset and the CVC-ClinicDB dataset, with a total of 1450 images combined from these two. The model is then tested on the remaining 10% unused subsample of the Kvasir-SEG and the CVC-ClinicDB dataset, as well as the other previously mentioned benchmark polyp datasets, ETIS, CVC-ColonDB, and EndoScene. A validation set is generated using a 10% subsample of the training data. The images in the Kvasir-SEG, CVC-ClinicDB, CVC-ColonDB, ETIS, and EndoScene datasets were randomly selected for input into the training, testing, and validation sets in correspondence with the aforementioned proportions. These divisions are tabulated in Table 1.

**Table 1:** Original dataset summary.

Dataset	Size	Total	Train	Valid	Test
Kvasir-SEG^8^	487 × 332 to 1920 × 1072	1000	900	90	100
CVC-ClinicDB^9^	384 × 288	612	550	55	62
CVC-ColonDB^10^	574 × 500	380	–	–	380
ETIS^11^	1225 × 966	196	–	–	196
EndoScene^12^	574 × 500	60	–	–	60

#### Training and implementation details

The model was developed using the PyTorch framework and was trained on 4 NVIDIA A100 GPUs. Image preprocessing applied to the input data was simple and standard. Images of polyps were resized from their native resolution to 512 × 512, and were then normalized using a mean ($\upmu$) and standard deviation ($\upsigma$) of 0.5 and 0.5, respectively. The performance was evaluated using the mIoU and mDice score.

The Adam optimizer was utilized for updating the learnable parameters in backpropagation, with a total number of epochs for training set to be 400. The batch size was set to 16. The learning rate was modified throughout training with a warm-up polynomial learning rate scheduler. The learning rate was started at an initial learning rate of $2.1052 \times {10}^{-5}$, which was then linearly increased until 20 epochs, and then was decreased using a polynomial degree of 0.98. Weight initialization values varied depending on the layer chosen. Convolutional and linear layers were initialized from a normal distribution ($\upmu =0$, $\upsigma =0.02$), layer normalization and batch normalization layers were initialized with weights and biases initialized to 1.0 and 0.0, respectively. The weights for the transformer encoder were pretrained on the ImageNet21k dataset to expedite performance increases during training and to avoid instability^39^.

## Results

The results of PSNet against previously performing SOTA models is presented through comparative studies. The primary purpose of these comparative studies are to demonstrate the current SOTA performance of PSNet. PSNet’s performance with respect to each of its newly developed components was also evaluated via ablation studies. The results from this analysis are presented in this section.

### Comparative studies

Using the training, validation, and test dataset configuration discussed in Table 1, our model generated an average mDice and mIoU score across all 5 datasets of 0.863 and 0.797, respectively. The corresponding values to each dataset are given in Table 2, along with previously SOTA models and their relative performance on each dataset as well. It can be clearly observed from the comparative studies results in Table 2 that PSNet’s performance is SOTA. The methods in the study use a wide variety of approach to polyp segmentation as discussed in the Introduction. For example, AMNet uses an input image size of 512 × 512, whereas TransFuse uses an input image size of 356 × 356. PraNet used a variable input image size into their network in which the input image was scaled by factors of {0.75, 1.0, 1.25} with respect to a base input image size of 356 × 356. Also note that the ViT architecture described in Table 2 consists of a ViT encoder with a partial decoder and dilated convolutional decoder.

**Table 2:** Results from comparative studies.

	Kvasir		ClinicDB		ColonDB		EndoScene		ETIS		Average	
	mDice	mIoU	mDice	mIoU	mDice	mIoU	mDice	mIoU	mDice	mIoU	mDice	mIoU
UNet	0.818	0.746	0.823	0.755	0.512	0.444	0.710	0.627	0.398	0.335	0.652	0.581
UNet ++	0.821	0.743	0.794	0.729	0.483	0.410	0.707	0.624	0.401	0.344	0.641	0.570
PraNet	0.898	0.840	0.899	0.849	0.709	0.640	0.871	0.797	0.628	0.567	0.801	0.739
ViT*	0.924	0.874	0.909	0.864	0.761	0.685	0.858	0.775	0.758	0.680	0.842	0.776
AMNet	0.912	0.865	0.936	0.888	0.762	0.690	–	–	0.756	0.679	0.842	0.781
TransFuse	0.92	0.87	0.942	0.897	0.781	0.706	0.894	0.826	0.737	0.663	0.855	0.792
Ours (PSNet)	0.929	0.879	0.928	0.879	0.795	0.715	0.877	0.802	0.787	0.713	**0.863**	**0.797**

Significant values are in bold.

As an additional comparative study, we developed our own training, validation, and test set by combining into a single set, the aforementioned datasets initially presented in Table 1 (i.e., the entirety of the Kvasir-SEG, CVC-ClinicDB, CVC-ColonDB, ETIS, and EndoScene datasets). The train, test, and validation split breakdown is shown in Table 3.

**Table 3:** Merged dataset train, validation, and test split breakdown.

Set	Train	Valid	Test
Number in dataset	2023	224	224

The primary purpose of this new study with a larger, merged dataset was to evaluate the issues associated with common datasets used and their configuration for training polyp segmentation models. As stated previously and found in our training procedure and in^15,16^, and^29^, the standard approach is to train the model on the Kvasir-SEG and the CVC-ClinicDB dataset. This approach, we argue negatively impacts the model’s ability to generalize and observe different polyp conditions by reducing its scope during training. Thus, we create a new dataset and modify its contents by merging all 5 publicly available datasets into a single set and randomly dividing them into a training, testing, and validation set. The contents were shuffled to contain a total of 2248 images and divided into a training, testing, and validation set, with 2023 train data images, and 224 images for the testing and validation set. These results provided a better combined mIoU and mDice score by increasing the model’s capacity to generalize. The maximum mIoU score reported on the test set was 0.897, and the maximum mDice score reported on the test set was 0.941 and are reported with respect to the original dataset performance metrics in Table 4.

**Table 4:** Comparative results of PSNet against old training procedure with original dataset configuration with the new training procedure with the modified merged dataset configuration.

Average across all 5 datasets	mDice	mIoU
PSNet’s results on the original dataset configuration	0.863	0.797
PSNet’s results on the new merged dataset configuration	0.941	0.897

### Ablation studies

A number of ablation studies were conducted to evaluate the model’s ability to segment images of polyps and evaluate the effects of increasing the model complexity on the model’s overall performance. The primary purpose of these studies is to measure the individual performance of each component. The ablation studies were conducted using the same train, test, and validation configuration as originally shown in Table 1. However, the reporting of the relative performance of each component was limited to the test data corresponding to the Kvasir-SEG and CVC-ClinicDB datasets to get a clear picture of each component’s impact on the model. We also follow the direction of other SOTA papers, such as in^15^, where only two datasets are used to conduct the ablation studies. The model was studied without the transformer encoder, without the additions of the dual decoder, without the merge modules, without the PS encoder and without CCM submodules in any of the LFE components in the PS encoder or decoder. These ablation studies are summarized in Table 5.

**Table 5:** Ablation studies.

Configuration of PSNet without:	Kvasir-SEG		CVC-ClinicDB	
	mDice	mIoU	mDice	mIoU
Transformer encoder	0.662	0.544	0.630	0.530
Dual decoder	0.817	0.729	0.867	0.787
Merge modules	0.876	0.815	0.890	0.839
PS encoder	0.908	0.854	0.908	0.864
CCM	0.916	0.865	0.916	0.867
PSNet (final)	0.929	0.879	0.928	0.879

For the ablation study of our transformer encoder, we remove the transformer encoder, and simply use the outputs from the PS encoder as input to the merge modules. Since the only outputs that the partial decoder and the enhanced dilated transformer encoder receive are the outputs from the transformer encoder, these are neglected as well. We notice the largest decrease in performance from the removal of this component. We observe a 28.7% decrease in the performance with respect to the mDice metric, and a 38.1% decrease in performance with respect to the mIoU metric. It follows that the largest decrease in performance of the model occurred with this component as this model was responsible for modeling global dependencies. This is a more expensive operation and carries more parameters. Thus, we would expect a larger decrease in performance. However, we note that our other components are still capable of modelling complex relationships.

In place of our dual decoder, we simply provide bilinear upsampling to the output of the dual encoder to obtain the segmentation map. With the removal of the dual decoder, we observe a significant decrease in the performance. With respect to the Kvasir-SEG dataset, we observe an 12.1% decrease in the mDice score and a 17.1% decrease in the mIoU score. We attribute this performance decrease to the loss of increased feature extraction that occurs with the dual decoder, both locally with the individual layers, and the loss of skip connections in the PS decoder.

We observe a decrease in the performance when removing the merge modules from the network. With respect to the Kvasir-SEG dataset, we observe a 5.7% decrease in the mDice score and a 7.3% decrease in the mIoU score. Clearly, with the introduction of the candidate segmentation maps produced by each of the merge modules, vital information both locally and globally is provided to the final segmentation map produced by the network.

With the removal of the PS encoder, we observe a decrease in performance. With respect to the Kvasir-SEG dataset, we observe a 2.3% decrease in the mDice score and a 2.8% decrease in the mIoU score. While clearly demonstrating its usage, we also note that within the dual encoder structure that the transformer component contributes more to the model performance. This makes sense intuitively given that the transformer component of the dual encoder focuses on long-range dependencies. However, we note that the introduction of the PS encoder is vital to refining the feature extraction necessary in the encoder to produce SOTA results.

We observe the smallest decrease in model performance with the removal of the CCM modules. With respect to the Kvasir-SEG dataset, we observe a 1.4% decrease in the mDice score and a 1.6% decrease in the mIoU score. While the smallest incremental increase across all ablation studies, this study demonstrates that these modules are essential to extract more rich semantic information by their wider receptive field.

We also conducted ablation studies about the parameter, μ, in an effort to understand the number of outputs of the transformer encoder and partial decoder on the performance of the model. These results are presented in Table 6.

**Table 6:** Ablation studies about μ across all datasets.

μ	mDice	mIoU
128	0.846	0.777
64	0.863	0.797
32	0.838	0.773
1	0.796	0.719

**Figure 6:**
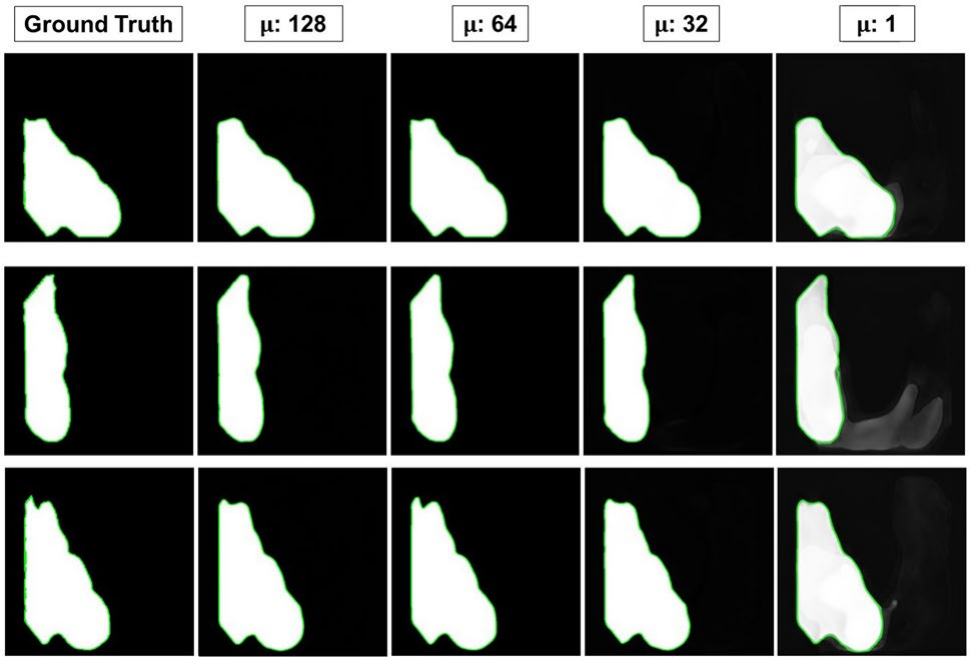
Boundary pixel lines drawn for a sample set of 3 input images. Each column represents the same PSNet model with different values of μ. Each row represents each of the 3 sample input images.

We observe the largest percent increase in performance with respect to μ between 1 and 32 and notice a smaller percent increase in performance with respect to μ between 32 and 64, we observe a decreasing trend at μ = 128. The maximum increase in performance studied was at μ = 64. Again, we attribute this increase in performance due to the transformer encoder being able to differentiate various types of pixels and allowing for an increase in learnable parameters as well. Figure 6 indicates the capacity of the parameter $\upmu$ to mine boundary pixel information by visualizing the outputs of images produced from the CVC-ClinicDB dataset. As can be seen from the figure, the boundaries get progressively less precise with decreasing values of μ. By increasing the value of μ, the model can better approximate corners and regions with more irregular shapes.

## Discussion

To demonstrate SOTA performance of our proposed network, we conducted extensive comparative studies using existing SOTA methods that were designed and/or applied to polyp segmentation. In particular, we examine the following networks, UNet^7^, UNet++^32^, PraNet^15^, AMNet^16^, and TransFuse^29^.

**Figure 7:**
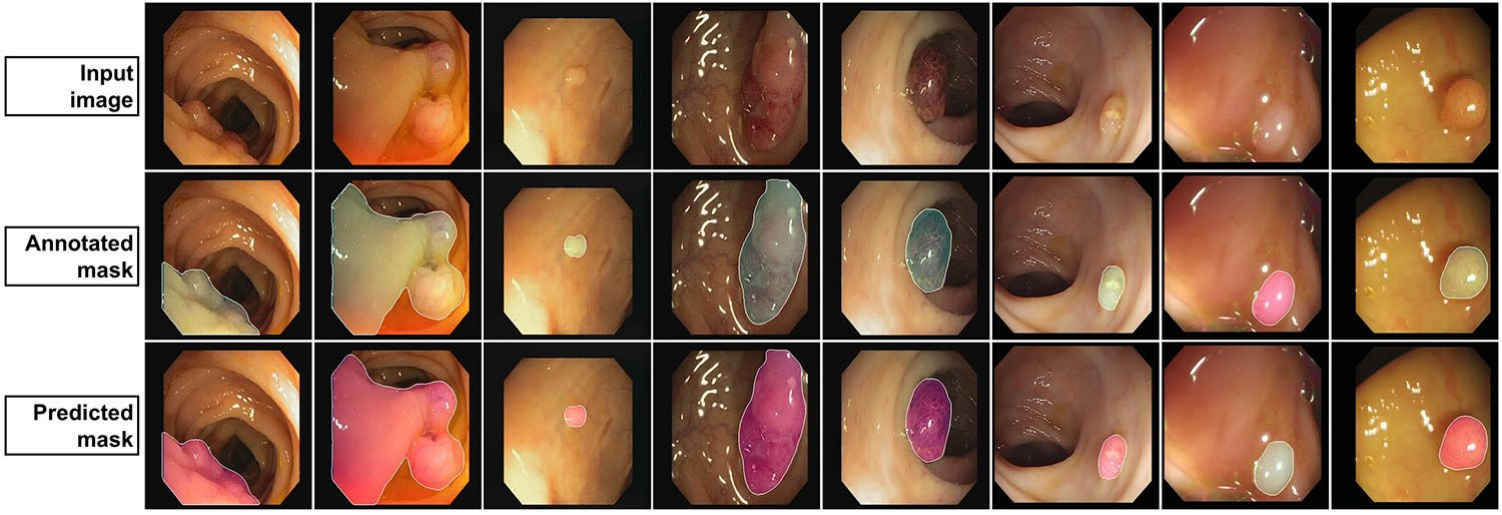
Sample model outputs with corresponding image input and ground truth.

Tables 2 and 4 summarize the best results for the model presented in this paper. Given the overall improvement in the combined mIoU and mDice score we deduce that our model generalization has improved and achieved SOTA and also improved upon boundary pixel identification. In particular, our model performed at an mDice of 0.863 and an mIoU of 0.797 across all 5 publicly available datasets evaluated. Over the course of 400 epochs, the best performance was obtained at epoch 218. Figure 7 summarizes 8 randomly selected images input into the model architecture (4 from unseen data and 4 from observed data), and shows the input image itself, as well as the corresponding ground truth and predicted segmented output.

Comparatively speaking, ETIS performed the worst in terms of performance metrics, as seen in Table 2. We attribute this due to the fact that the polyps in this dataset are smaller on average and have generally different textures and colors contrasted to the training set. Broadly speaking, the model characterized larger polyps better than smaller polyps. Conversely, the Kvasir-SEG and CVC-ClinicDB dataset perform the best, which makes sense intuitively given that they constituted the entirety of the training set. In general however, the segmented results are highly accurate and our model shows excellent generalization capabilities, thus showing clear industry application and performance capabilities for clinical settings.

We argue that the approach of merging the respective datasets used in this study (i.e., Kvasir-SEG, CVC-ClinicDB, CVC-ColonDB, EndoScene, and ETIS) to produce a new merged set is a better approach to the polyp segmentation problem in terms of data collection and evaluation. This is because limiting the training set to only the Kvasir-SEG and CVC-ClinicDB dataset reduces the model’s ability to be exposed to different environmental conditions present in clinical settings. The model is limited in the sense that the Kvasir-SEG and the CVC-ClinicDB dataset demonstrate a specific camera setup, lighting condition, and patient sample. Training the model on only these datasets causes the model to lose its ability to generalize. By randomly shuffling the entirety of available polyp segmentation sets used in this paper (i.e., also including the CVC-ColonDB, EndoScene, and ETIS datasets) and generating a new set, we show that model performance is actually being underrepresented in SOTA models while also presenting flaws in the approach to training. This is clearly evident by our higher mIoU and mDice score reported on the new merged set (mIoU: 0.897, mDice: 0.941), compared to the average mIoU and mDice score reported on the original training set in the comparative study (mIoU: 0.797, mDice: 0.863). Thus, the model shows increased ability for generalization. Comparing these results to the reported results of other models, we observe a 10% increase in the mIoU score, and an almost 8% increase in the mDice score. Given that the included datasets have a more diverse range of polyps present in the image samples, we can see that the prospect of expanding the training set to include polyps of a wider variety of shapes, colors, and sizes, has a significant effect on the accuracy of the model.

## Conclusion

In an effort to tackle the issues associated with boundary pixel isolation and improvement, modelling of global dependencies and context, as well as model generalization issues extant in existing networks in the field of polyp segmentation, we proposed our novel end-to-end dual encoder–decoder architecture, PSNet. Both the dual encoder and decoder were developed by the careful incorporation of a variety of deep learning technologies, including the PS encoder, transformer encoder, PS decoder, the enhanced dilated transformer decoder, the partial decoder and the merge module. PSNet demonstrated SOTA performance (in terms of mDice and mIoU) through extensive comparative studies using 5 existing polyp datasets. Using the standard dataset with Kvasir and CVC-ClinicDB datasets for training, we obtained an mDice and an mIoU score of 0.863 and 0.797 respectively. Using our new modified dataset we obtained an mDice and an mIoU score of 0.941 and 0.897 respectively.

## Data Availability

The datasets utilized in this research study, such as the Kvasir-SEG and the CVC-ClinicDB dataset can be individually found here and here, respectively. The specific training and test dataset configuration can be found here and here, respectively.
